# Self-rated health as a valid indicator for health-equity analyses: evidence from the Italian health interview survey

**DOI:** 10.1186/s12889-019-6839-5

**Published:** 2019-05-09

**Authors:** Beniamino Cislaghi, Cesare Cislaghi

**Affiliations:** 1 Department of Global Health and Development, London School of Hygiene and Tropical Medicine, 15-17 Tavistock Place, London, WC1 9SH UK; 2 Agenzia Nazionale per i Servizi Sanitari Regionali, Rome, Italy

**Keywords:** Self-rated health, Equity, National Health Service, Health policy, Healthcare demand, Italy, Self-rated health as a valid indicator for health-equity analyses: evidence from the Italian Health Interview Survey

## Abstract

**Background:**

Self-rated health is widely considered a good indicator of morbidity and mortality but its validity for health equity analysis and public health policies in Italy is often disregarded by policy-makers. This study had three objectives. O1: To explore response distribution across dimensions of age, chronic health conditions, functional limitations and SRH in Italy. O2: To explore associations between SRH and healthcare demand in Italy. O3: To explore the association between SRH and household income.

**Methods:**

Cross-sectional data were obtained from the 2015 Health Interview Survey (HIS) conducted in Italy. Italian respondents (*n* = 20,814) were included in logistic regression analyses. O1: associations of chronic health conditions (CHC), functional limitations (FL), and age with self-rated health (SRH) were tested. O2: associations of CHC, FL, and SRH with hospitalisation (H), medical specialist consultations (MSC), and medicine use (MU) were tested. O3: associations of SRH and CHC with household income (PEI) were tested.

**Results:**

O1: CHC, FL, and age had an independent summative effect on respondents’ SRH. O2: SRH predicted H and MSC more than CHC; age and MU were more strongly correlated than SRH and MU. O3: SRH and PEI were significantly correlated, while we found no correlation between CHC and PEI.

**Conclusions:**

Drawing from our results and the relevant literature, we suggest that policy-makers in Italy could use SRH measures to: 1) predict healthcare demand for effective allocation of resources; 2) assess subjective effectiveness of treatments; and 3) understand geosocial pockets of health inequity that require special attention.

## Background

In spite of international consensus on the validity of Self-rated Health (SRH) as a good predictor of morbidity and mortality [1–5], health policy-makers in Italy have disregarded SRH measures to shape health-related policies, predict healthcare demand and run health equity analysis. Using data from the 2015 Health Interview Survey (HIS), this study investigates in particular whether SRH measures can be a valuable indicator for health equity analysis in the Italian national health service and potentially elsewhere too.

Several health population surveys include one or multiple questions on self-rated health (SRH) [2]. There is little doubt that the question used to measure SRH is meaningful to respondents [6, 7]. Evidence also exists that self-rated health is a stable concept, as it is formed in adolescence and remains highly consistent throughout adulthood so that people express the same opinion on their health when experiencing the same internal feelings [8].

However, in spite of these and more studies showing that SRH can accurately predict mortality and morbidity, its validity is still contended [9]. Part of the controversy originates from the debate on whether SRH is an accurate measure of an objective health status. SRH measures have been under the severe scrutiny of those arguing that one’s understanding of their health (the “internal” view) might be very misaligned with the opinion of medical experts (the “external” view) [10]. Questions have been raised, for instance, on how psychological traits such as a fatalism or hypochondria [11, 12], larger cultural values and social norms [13, 14] or gender [15–17] might both influence responses to SRH questions, and limit the potential of SRH measures for cross-country comparisons. SRH measures can also sometimes show erratic trends in short-term analysis, as sometimes the data rely on a limited number of respondents, although useful insights can come from examining datasets collected over multiple years [10]. However, much of the available research suggests that the two (SRH and objective health status) are certainly related. In a seminal study, Blaxter [18] found that self-reported chronic conditions and diagnosed chronic conditions overlapped in 80% of the cases. More recently, using a large sample from five major Chinese cities, Wu and colleagues [19] found SRH to be largely consistent with objective health status. And before then, Haddock and colleagues [20] came to a similar conclusion on the validity of SRH analysing a cross-sectional health survey of military personnel. Other SRH-related measures, such as self-reported functional limitations, have also been found to be fairly accurate, especially when respondents have physical (as opposed to mental and social) limitations [21] and have been increasingly used to inform public health resource allocation policies [22].

Other commentators, however, have argued that the debate on SRH should not focus on whether it overlaps with diagnosed diseases. Rather, they have suggested that the discussion on the use of SRH is an ethical one, that relates to the goal of a national public health system and, potentially, to the very definition of what health is. Waller [23], for instance, argued that SRH is an extremely valuable measure of health as it measures what really matters: “Doctors can liberate patients and empower them to health rather than oppressing them with diagnosis, risk factors, and seeing problems. Focusing on self-rated health can help to empower patients” (p.110). One might go as far as to argue that it would be better for people to feel well, despite the presence of pathological conditions, than for them to feel bad in the absence of those conditions. The health system should thus increase its capacity to help people feel that “things go well with them” [24]. If a medical diagnosis cannot help to improve how one person feels or will feel in the future, why would that diagnosis be necessary? That is, why should the health system increase service demand of the people who feel well (beyond prevention purposes)? If people feel healthy, a therapy should only be necessary to prevent the worsening of their SRH in the future. Such an approach to health and healthcare – fairly controversial among Italian public health policy makers – is not new: Marinker [25] famously (and revolutionarily) asked over twenty years ago: why should we make people patients? And, more recently, Misselbrook, Dean Emeritus of the Royal Society of Medicine, suggested that the health system should focus more on helping people be well, beyond just telling them why they are sick [26]. In this paradigm of health, where subjective health matters as the ultimate goal, the health system would need to be more concerned with people’s illnesses, the experience of “unhealth … interior to the person of the patient,” rather than largely focussing on their diseases, the “pathological processes, most often physical … [associated with] some deviation from a biological norm” [27].

### Study objectives

This paper offers insights into whether SRH can be a valuable indicator for designing effective public health policies on population health and equitable access to the national health service. Data from the 2015 Health Interview Survey (HIS) were used (more details in the methods section). Three objectives, specifically, guided our analysis.

Objective 1: *To explore response distribution across dimensions of age, chronic health conditions, functional limitations and SRH in Italy*. The existing evidence shows great differences in what is associated with SRH, ranging from, for instance, physical exercise in Sweden [28], religiosity in the Caribbeans [29], education in Ireland [30], gender in Lithuania [31], and social capital and optimism towards life in Portugal [32]. While some evidence exists on what drives SRH in other European countries, little is known about what contributes to SRH in Italy. We looked specifically at the association between age, chronic health conditions, functional limitations and SRH, using key indicators available in the dataset to understand the independent effect of diseases (objective diagnosis of a chronic health condition) and sicknesses (functional limitations) on respondents’ subjective feeling of wellbeing, while also testing the effect age as, possibly, the most important explicator factor of participants’ health.

Objective 2: *To explore associations between SRH and healthcare demand in Italy*. Nothing in the literature exists on the validity of SRH as a measure to predict the use of healthcare services for effective resource allocation in Italy. The international literature, instead, includes some important studies on this very issue (e.g. [33–35]). A study by Hunt and colleagues, for instance, suggested that SRH measures could be a better predictor of utilization of UK health services than mortality and morbidity statistics [36], and in another study conducted in Finland, the authors found SRH to be significantly predictive of healthcare demand [1]. Within the literature on the Italian health system, age and chronic health conditions are often considered optimal proxies for predicting the volume of services provided, as they are found to be strongly correlated with healthcare demand [37], but, as we mentioned, there is no reference to SRH in such literature.

Objective 3: *To explore the association between SRH and household income*. The association between SRH and income has been proven across several countries. Commentators suggested that they are positively correlated because money increases healthcare options and resource-deprived people often experience other forms of social inequalities that can result in social and emotional deprivation [38–41]. No evidence on the association between SRH and income in Italy is available in the international literature, yet it is critical to explore this association for policy-makers to decide whether to integrate SRH within health equity analyses to inform more equitable policies.

## Methods

### Sample

We used data from the Health Interview Survey (HIS) designed by EUROSTAT and conducted by ISTAT in Italy in 2015. The sample is derived from a multistage probability sample of households. The sample includes population aged 15 or over living in the territory of the country [42]. The survey includes four modules: 1) health status, 2) health care use, 3) health determinants and 4) socio-economic background variables [43]. (The full questionnaire is available at https://ec.europa.eu/eurostat/documents/3859598/5926729/KS-RA-13-018-EN.PDF/26c7ea80-01d8-420e-bdc6-e9d5f6578e7c). The percentage of those in age 15–29 not reporting good or very good health is very low (see Table 1). We decided thus to exclude this part of the sample to avoid unbalancing our analysis. Our sample thus included 20,814 men and women aged 30 or more.

**Table 1:** Number of respondents and percentage in the sample reporting fair, bad or very bad self-rated health by age group

Age	Sample	Report fair/bad/very bad health
15–29	4301 [16.9%]	272 [3.2%]
30+	20,814 [83.1%]	7394 [96.8%]
Total	25,325 [100%]	8500 [100%]

### Variables

Outcome variable was Self-Rated Health, measured in the HIS using the standardised question created by the WHO: “*How is your health in general? Very good; Good; Fair* [translated in Italian as ‘neither good or bad’]*; Bad; Very bad*” (question code: HS1).

We used different predictor variables for each objective. Objective 1. Covariates used in our analyses for objective 1 include age, chronic health conditions (HS2: *Do you have any longstanding illness or [longstanding] health problem?*), functional limitations (HS3A: *Are you limited because of a health problem in activities people usually do? Would you say you are… severely limited; limited but not severely; not limited at all)* and 28 diagnosed health conditions.

Objective 2. Covariates used for objective 2 included hospitalisation (H02B: *In the past 12 months (prior to the survey interview) how many times have you been admitted to hospital as a day patient?*), consultation of medical or surgical specialist (AM5: *During the past four weeks, how many times did you consult a specialist on your own behalf?*) and medicine use (MD1: *During the past two weeks (prior to the interview), have you used any medicines that were prescribed for you by a doctor?*).

Objective 3. Covariates used for objective 3 included age, sex, geographical residence and “personal equivalent income” (PEI). PEI is an indicator obtained dividing household disposable income by the number of household members [44, 45]. In our analysis, we used PEI quintiles to create a dichotomous variable. In the first group (higher PEI) we included individuals within the highest two PEI quintiles (40% of the entire sample) and in the second group (lower PEI) we included individuals in the remaining three PEI quintiles (60%).

### Statistical methods

Several logistic regression models and descriptive cross-tabulations were used. The dependant variable is the SRH response converted into a dichotomous variable: *better* (reports of very good and good health) and *worse* (reports of fair, bad, and very bad health). As others did before (e.g. [46, 47]), we created a dichotomous variable, having observed stark differences in the Italian sample across the two groups of better and worse health, but less important differences within the two groups. The HIS in Italy allows response by proxy (e.g. a family member responding to the questions for someone incapacitated to do so), which reduces presence of missing data. Despite this, there were 210 missing observations in the dataset for the SRH question (1% of the total sample). We removed these 210 from our sample. For objective 1, we used a simple cross-tabulation to describe the association between SRH, age, and chronic health conditions, and, separately, at the association between SRH, age, and functional limitations. For chronic health conditions there were 30 missing responses (0.15% of total responses); for functional limitations there were 525 missing responses (2.5% of total responses). For objective 2, we conducted a logistic regression to test the association between SRH, age, and chronic health conditions with hospitalisation, medical examination, and prescriptions. Finally, for objective 3 we conducted a logistic regression to test the association between SRH and PEI, weighting PEI by region of residence. In all analyses, where appropriate, we controlled for age, income, sex, and region of residence.

## Results

### Descriptive data

Table 2 reports descriptive data on the sample included in this analysis.

**Table 2:** Descriptive data for participants in the sample

	Observations	Percentage in the Sample
Sex		
Male	9817	47.2%
Female	10,997	52.8%
Age		
30–34	1627	7.8%
35–39	1746	8.4%
40–44	2208	10.6%
45–49	2301	11.1%
50–54	2266	10.9%
55–59	2013	9.7%
60–64	1903	9.1%
65–69	1883	9.0%
70–74	1491	7.2%
75–79	1357	6.5%
80–84	1040	5.0%
85+	979	4.7%
Region of residence		
North-west	5161	24.8%
North-east	4412	21.2%
Centre	4182	20.1%
South	4913	23.6%
Islands	2146	10.3%
PEI quintiles		
1 (Poorest)	3566	17.0%
2	4013	19.1%
3	4250	20.2%
4	4515	21.5%
5 (Richest)	4680	22.3%
Better SRH		
Very good	3295	15.8%
Good	10,125	48.6%
*Total better SRH*	*13,420*	*64.5*%
Worse SRH		
Fair	5313	25.5%
Bad	1638	7.9%
Very bad	443	2.1%
*Total worse SRH*	*7394*	*35.5*%
Total	20,814	**100%**

## Main results

### Association between age, chronic health conditions, functional limitations and SRH

Table 3 reports results of the cross-tabulation used to describe the association between SRH and chronic health conditions, and the association between SRH and functional limitations.

**Table 3:** Response distribution percentage for self-rated health and chronic health conditions, and for self-rated health and functional limitations

Self-rated health	Chronic health conditions		Functional limitations		
	Cohen’s K = .502 p < 0.001		Cohen’s K = .349 *p* < 0.001		
	Yes	No	Severe	Non-severe	No
Better	33.1%	82.9%	8.6%	27.7%	82.8%
(very well, well)	(5.137)	(2.247)	(1.763)	(3.128)	(2.412)
Worse	66.9%	17.1%	91.4%	72.3%	17.2%
(fair, bad, very bad)	(7.675)	(13.109)	(1.929)	(1.929)	(14.023)

**Fig. 1:**
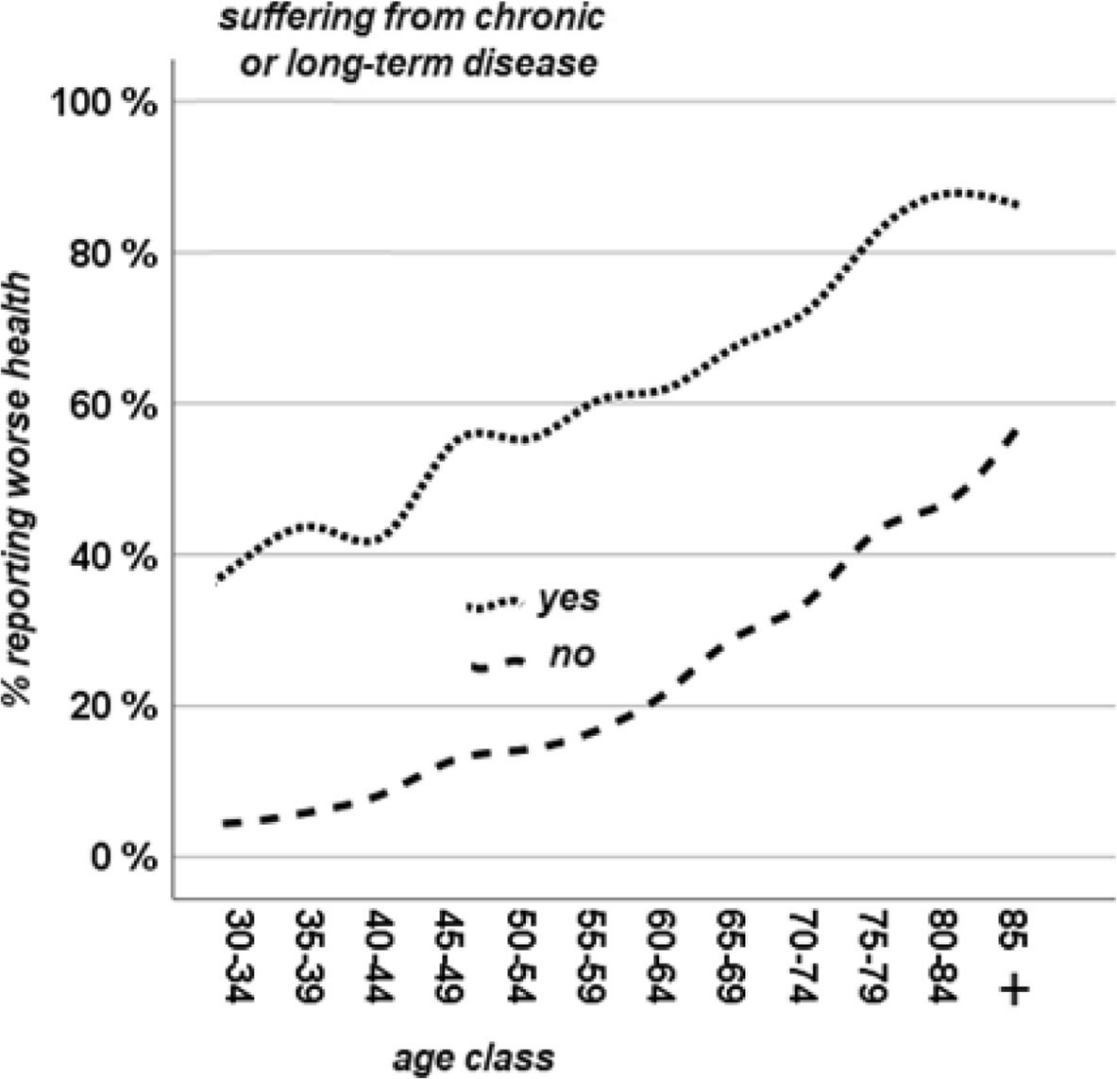
% reporting worse health by age and chronic health conditions

Among those who declared to have a chronic health condition, 33.1% reported better health as compared to only 8.6% of those with severe functional limitations. Data examined by age cohorts show that both those respondents affected and unaffected by chronic health conditions are more likely to report worst health as their age increases (see Fig. 1).

**Fig. 2:**
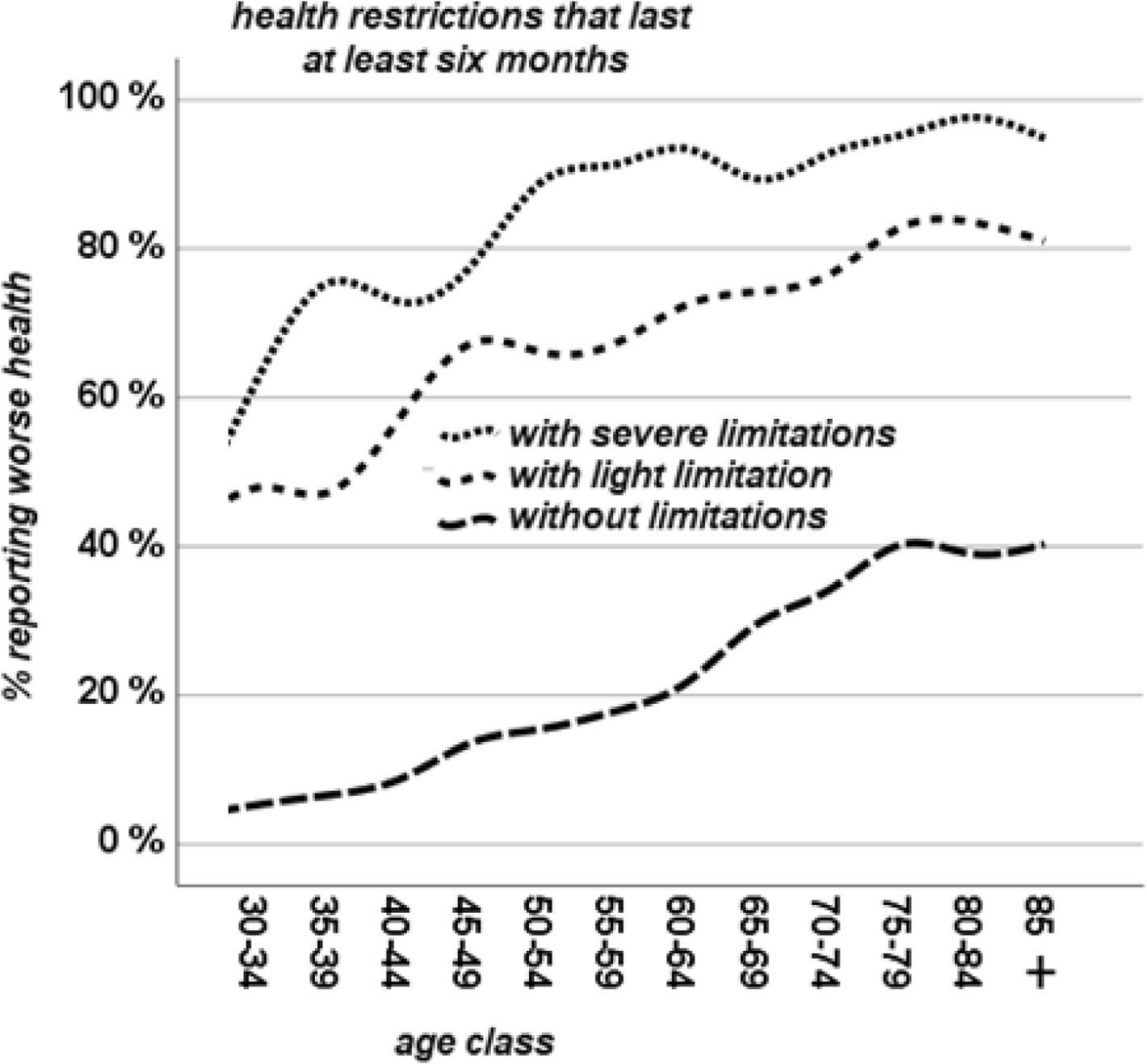
% reporting worse health by age and limitations

Figure 1 further shows that age acts independently as a contributor to worse SRH. While approximately 40% of 30/34-years-olds with chronic health conditions report worse health, this percentage increases to more than 80% among those aged 85 and above with chronic health conditions. At the same time, respondents’ SRH worsened with age at a similar trend for both those with and without a CHC. The same is true for functional limitations: respondents’ SRH became worse as they aged, independently of the severity of their limitations. (see Fig. 2).

**Fig. 3:**
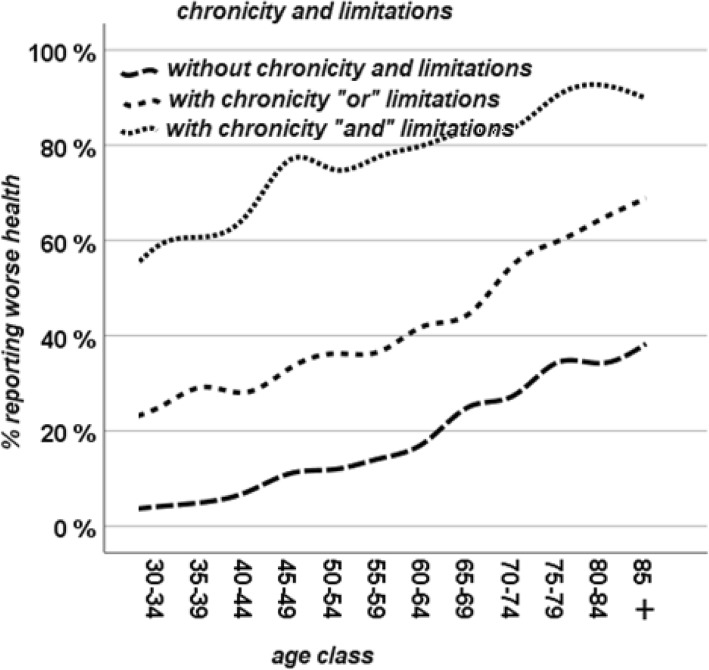
% reporting worse health by age and chronic health conditions and/or functional limitations

Figure 3 shows the association of any chronic health conditions and any limitations with SRH.

Having both a chronic health condition and a limitation has obviously the worst effect on SRH. However, similarly to what observed in Figs. 1 and 2, age had an independent effect on SRH, not modified by chronic health conditions (Fig. 1), functional limitations (Fig. 2) or both (Fig. 3). Chronic health conditions, functional limitations, and age have summative effect on each other, acting independently in how they influence SRH.

**Fig. 4:**
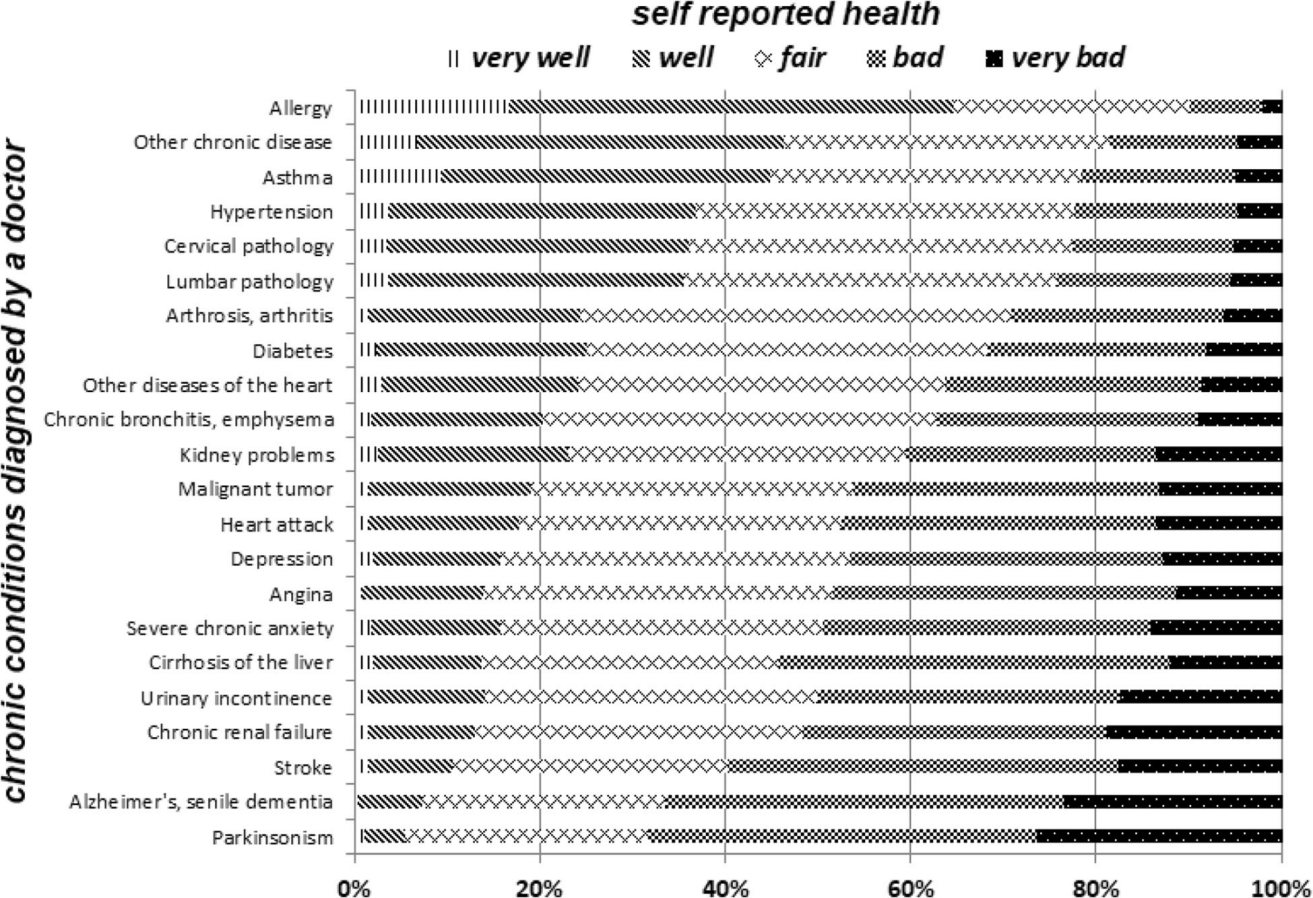
Self-rated health by diagnosed chronic health conditions

**Fig. 5:**
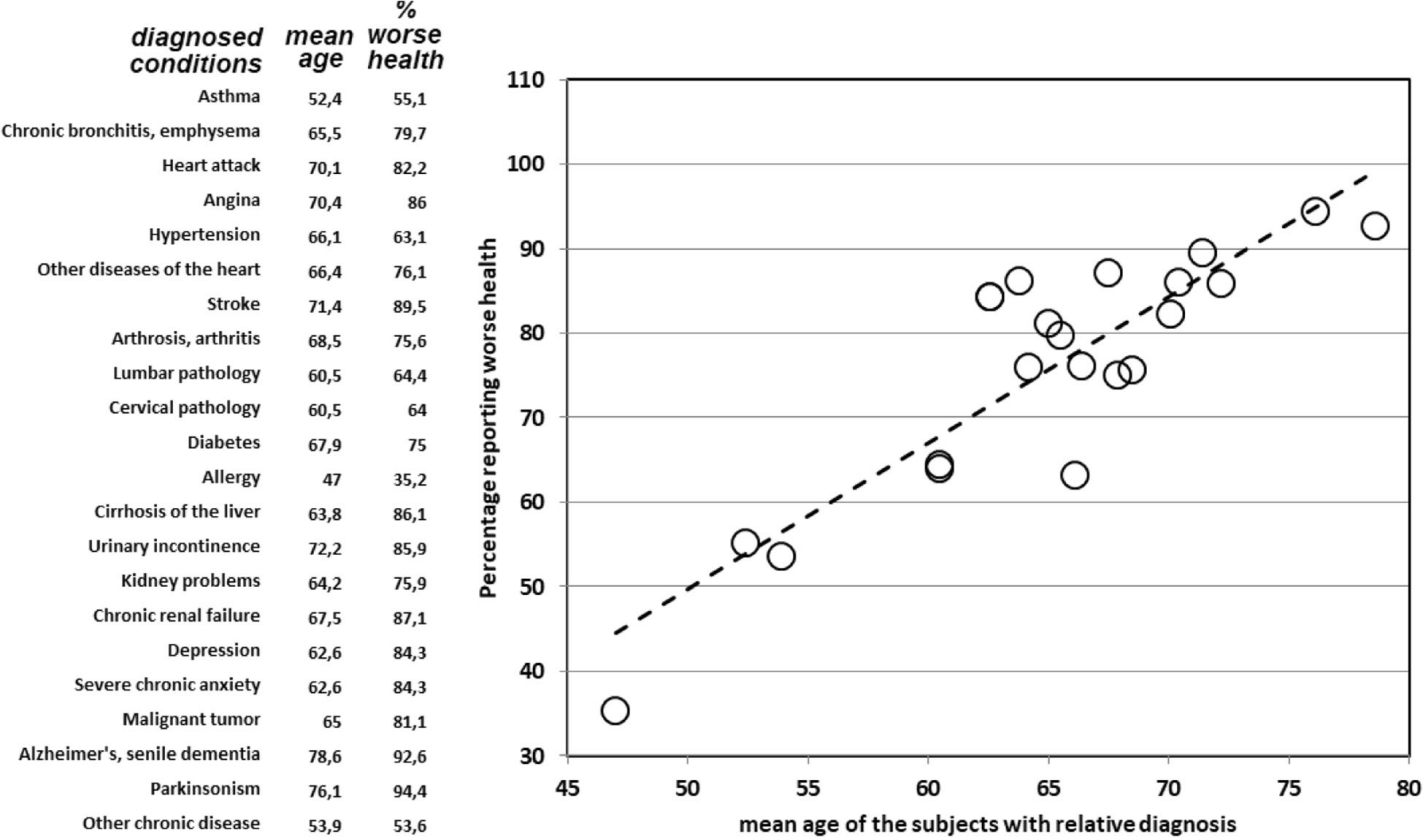
Average age for population diagnosed with each medical condition, and % with worse SRH

We looked further at the effect of SRH and health conditions. Figures 4 and 5 show, respectively, the effect that specific diagnosed health conditions have on SRH and the results of the linear regression between average age of respondents with a diagnosed health condition and the percentage of respondents with worse SRH.

**Fig. 6:**
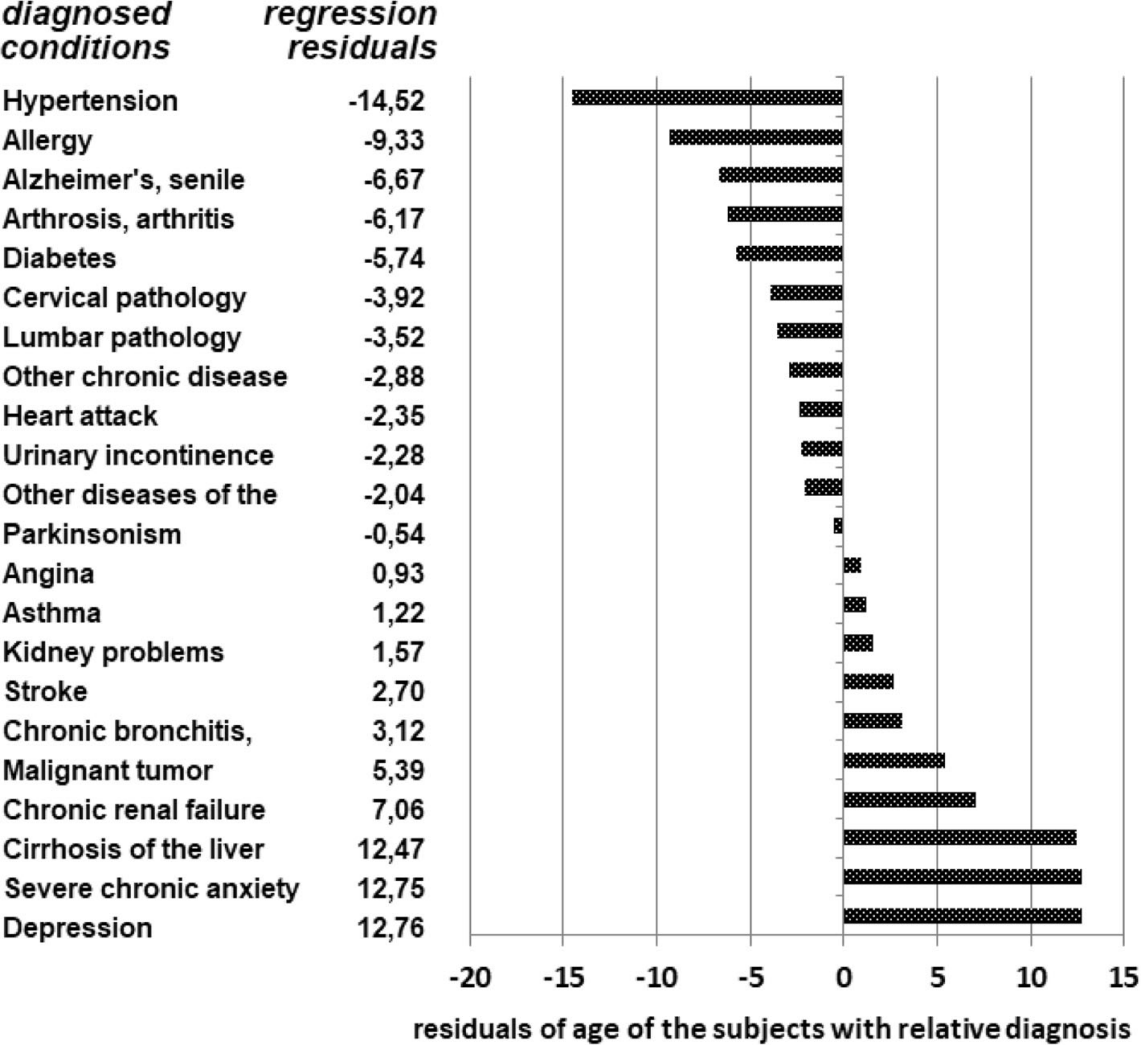
Regression residuals

Age explains ¾ of the variability in the percentage of respondents reporting worse health (R2 = .77). Alzheimer’s disease is the condition with the highest average age (92.6% reporting worse health), while allergies are the condition with the lowest average age (35.3% reporting worse health). Due to the fact that certain health conditions are more likely at different ages (for instance, it is more likely to develop hypertension at older age) it was important to run the regression residuals presented in Fig. 6.

As shown in Fig. 6, there is some variability across health conditions in the extent to which age determines SRH. The Figure ranks regression residuals for each health condition. For instance, in the case of hypertension, allergies, and Alzheimer’s disease, the health condition affects respondents’ SRH less than their age, when compared to the regression model. In the case of depression, chronic anxiety, and hepatic cirrhosis, instead, the health condition affects respondents’ SRH more than their age, when compared to the regression model. Even taking into account this variability, the regression model suggests that, overall, worse SRH is affected more by respondents’ age than by their chronic conditions.

### Associations between SRH and healthcare demand

Table 4 shows the result of the logistic regression testing the associations of SRH, age, chronic health conditions with 1) the number of hospital admissions in the last year (prior to the survey interview); 2) the number of medical specialist consultations in the last four weeks (prior to the interview); and 3) the drugs prescribed in the last two weeks (prior to the interview).

**Table 4:** Odd ratios of having received at least one medical consultation in the last month, have been hospitalised in the last 12 months, or have received drug prescription in the last 15 days

	Received at least one medical specialist consultation in the four weeks			Hospitalized at least once in the last year			Received drug prescriptions in the last two weeks		
	OR	95% C.I.		OR	95% C.I.		OR	95% C.I.	
		lower	upper		lower	upper		lower	upper
Chronic Diseases									
Asthma	1199	1194	1203	1132	0,921	1391	1255	1067	1476
Chronic bronchitis	1267	1262	1271	1204	1010	1437	1152	0,986	1347
Myocardial infarction	1490	1483	1498	1981	1585	2477	1879	1450	2434
Angina pectoris	1275	1269	1281	1642	1332	2024	1019	0,814	1277
Hypertension	1200	1198	1202	1005	0,894	1129	2692	2484	2917
Stroke	0,904	0,899	0,909	1636	1263	2121	1007	0,751	1350
Arthrosis and arthritis	1131	1129	1133	0,940	0,827	1067	1203	1097	1320
Diabetes	1374	1370	1377	1111	0,955	1292	1503	1321	1711
Cirrhosis of the liver	0,587	0,579	0,595	1898	1054	3421	0,978	0,505	1893
Kidney failure	1246	1239	1254	1576	1174	2115	1162	0,834	1619
Depression	1455	1449	1460	0,875	0,709	1079	1709	1413	2067
Chronic anxiety	0,902	0,898	0,906	1339	1064	1685	1252	0,999	1570
Malignant tumour	2567	2556	2579	3126	2547	3837	1724	1375	2162
Alzheimer’s disease	0,756	0,751	0,761	0,883	0,660	1181	1244	0,911	1699
Parkinson’s disease	0,972	0,963	0,981	0,764	0,485	1203	1149	0,702	1882
Self-rated health									
Very well	0,635	0,633	0,637	0,500	0,397	0,630	0,509	0,459	0,564
Well	1	–	–	1	–	–	1	–	–
Fair	1681	1678	1684	1981	1744	2251	1734	1603	1875
Bad	3105	3095	3114	4254	3601	5026	2310	1997	2671
Very bad	2455	2442	2467	6087	4756	7791	2352	1795	3082
Demographics									
30–34	1	–	–	1	–	–	1	–	–
35–39	0,995	0,991	0,999	1411	1026	1941	1040	0,876	1235
40–44	0,970	0,966	0,974	1114	0,814	1524	1203	1025	1412
45–49	0,974	0,970	0,978	1158	0,854	1571	1302	1112	1523
50–54	1057	1053	1061	1037	0,762	1410	1447	1237	1693
55–59	0,941	0,937	0,945	1204	0,888	1633	1805	1539	2118
60–64	0,973	0,969	0,977	1448	1072	1956	2211	1880	2600
65–69	1018	1014	1022	1248	0,920	1692	2338	1981	2759
70–74	1033	1029	1038	1573	1156	2140	2567	2153	3061
75–79	0,970	0,966	0,975	1456	1067	1988	2692	2237	3240
80–84	1067	1062	1072	1714	1247	2356	2669	2181	3268
85 +	0,761	0,757	0,764	1782	1291	2460	2695	2179	3333
Sex									
Male	1	–	–	1	–	–	1	–	–
Female	1396	1394	1399	0,931	0,841	1031	1299	1219	1384
Geographic area									
North west	1	–	–	1	–	–	1	–	–
North East	1022	1020	1025	1025	0,887	1183	1153	1053	1263
Centre	0,996	0,994	0,999	0,937	0,809	1085	1019	0,928	1118
South	0,693	0,692	0,695	0,798	0,690	0,922	0,865	0,790	0,947
Islands	0,814	0,811	0,816	0,960	0,805	1146	0,750	0,667	0,844

As reported in Table 4, SRH has the highest odd ratios for each of factors 1 and 2 mentioned above (with the exception of factor 3, as discussed below). More in detail, Table 4 shows that, when controlling for other factors, age played a limited role in affecting hospital admissions. The health conditions most likely to result in people’s hospitalisation are cancer, cirrhosis, previous infarction, and stroke. Even though health conditions are associated with seeking hospitalisation, however, worse SRH bears the strongest association with hospitalisation than any other variables.

In the second column, Table 4 reports the factors affecting respondents’ access to medical specialist examinations in the four weeks prior to the survey interview. Again, SRH is the variable that affects the most respondents’ decisions to request a medical specialist examination. Interestingly, respondents with bad SRH show higher odds to access medical examinations than respondents reporting very bad SRH (possibly as the latter are already familiar with their health condition). As for the role played by diagnosed chronic health conditions, the only one that increases respondents’ odds to access medical examinations are tumours.

The third column of Table 4, finally, reports odd ratios for having received a drug prescription in the last two weeks. Here, age plays a larger role than in the previous two. This is probably because, in older people, the number of prescribed drugs likely accumulates over time. Even though age affects significantly the likelihood of receiving a drug prescription, SRH still bears higher odd ratios than the diagnosed presence of chronic health conditions.

### Association between SRH and PEI

**Fig. 7:**
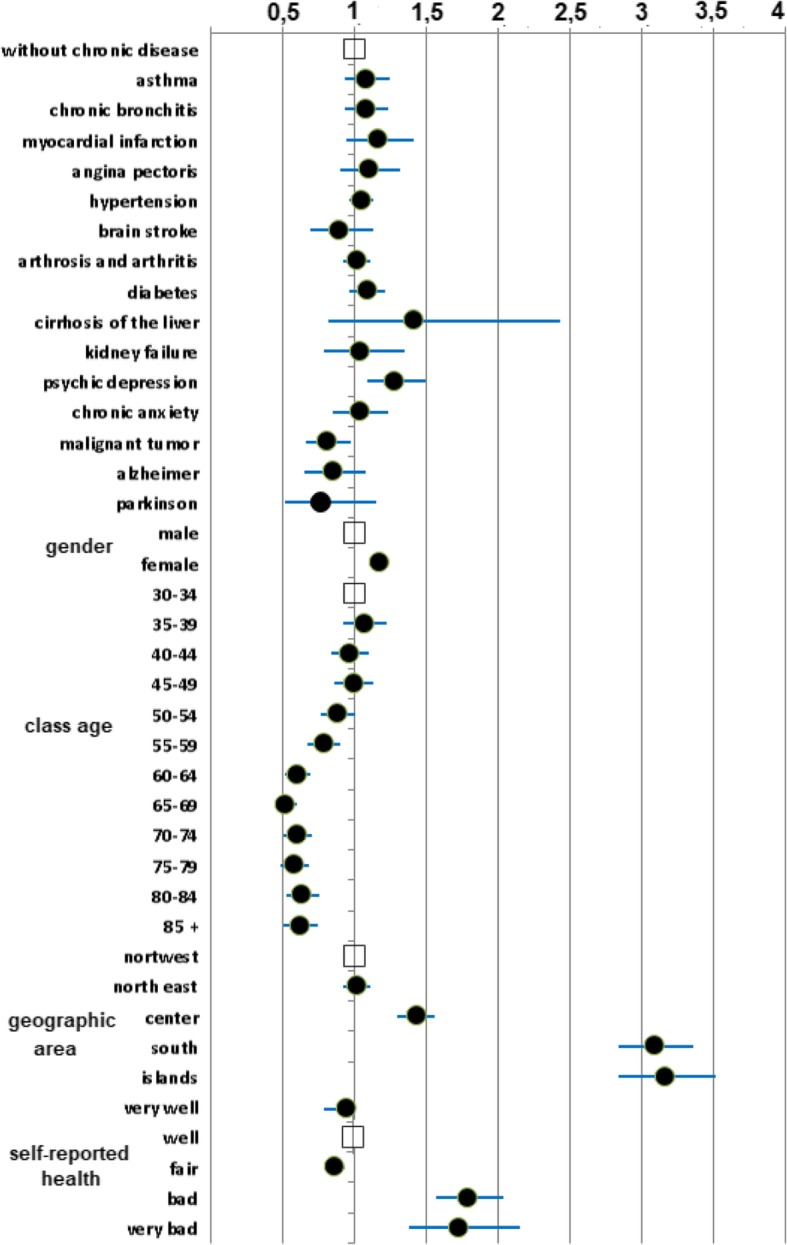
Logistic regression of the risk of being in the lowest two income quintiles by age, sex, geographic area, chronic health conditions, and self-rated health

HIS data reveal two possible confounders of income: region of residence and age. People living in the North-west of the country are 3 times more likely to be in the two highest PEI quintiles when compared to people living in the Southern or Islands regions. People aged 40–70 are 2 times more likely to be in the two highest PEI quintiles than other people, with men being at slighter greater advantage over women of the same age (data not shown). Informed by this analysis, our logistical regression model tests the joint effect of age, sex, geographical residence, chronic health conditions, and SRH on the probability of being in the lowest two PEI national quintiles. Figure 7 shows the results of this regression.

As shown in Fig. 7, the risk of having a PEI in the lowest two quintiles is not significantly different for people with any of the chronic health conditions included in Fig. 7 (with the exception of depression) when compared to people without any of those conditions. Conversely, the risk of having a PEI in the lowest two quintiles is significantly higher for those reporting worse SRH.

## Discussion

We set up this study with three objectives. Objective 1 was “*to explore response distribution across dimensions of age, chronic health conditions, functional limitations and SRH in Italy”*. We found that age, chronic health conditions, and functional limitations had an independent summative effect on SRH. Objective 2 was “*to explore associations between SRH and healthcare demand in Italy”*. We found that, even though age affects significantly the likelihood of receiving a drug prescription, SRH predicted healthcare demand more than the diagnosed presence of chronic health conditions. Objective 3 was: “*to explore the association between SRH and income”*. We found that SRH increased the risk of having a lower PEI more than the presence of a chronic health condition. We hypothesis this difference to be due to the fact that people with a lower PEI might struggle more to compensate for their chronic health condition. That is, we suggest that the experience of a condition (rather than the condition itself) affects more the SRH of poorer than that of richer people.

We suggest three critical implications for health policy-makers in Italy and possibly Europe at large following our result as contextualised in the relevant literatures. These implications are related to using SRH measures to: 1) predict healthcare demand for effective allocation of resources; 2) assess subjective effectiveness of treatments; and 3) understand geosocial pockets of health inequity that require special attention.

Whether SRH can be used as a predictor of service utilisation has been the subject of an intricate and unresolved debate. Some have argued that SRH measures in equations for the utilisation of healthcare are endogenous; that is, respondents might be more likely to rate their health bad if they have recently visited a doctor [22] and, for this reason, that SRH cannot be used as a prediction measure (but, rather, as post-diction measures, as people’s SRH might be dependent on having visited the health service before the survey) [34]. This is a valid objection, yet the level of bias produced by the potential endogeneity of the indicator should be carefully assessed, especially since other studies have provided evidence of the usefulness of using SRH to this predictive purpose. Our findings suggest that, in 2015 in Italy, hospitalization and specialist consultations were generally less influenced by the presence of an actual disease than by people’s belief of being in a poor health state. Integrating SRH status survey instruments within studies would help the Italian national health system allocate resource more effectively, contributing to predicting the volume of future medical consultations.

Treatment effectiveness evaluations in Italy tend to focus on objective clinical measures of disease. This preference is due to both the fact these measures are often considered easier to quantify and interpret, as well as the fact that self-rated measures do not have the same aura of “medical expertise” [48]. A considerable body of literature is emerging on the importance of integrating measures of patient reported experience and patient reported outcomes as part of a National Health System effectiveness evaluations [49–51]. Drawing from that literature, we suggest that SRH measures can be critically important for the Italian national health system, as they allow to measure what matters to the people that the system is supposed to serve: how these people feel. Clinical diagnostic practices are used to compare objective measures (as, for instance, laboratory exams) with subjective accounts of one’s medical history. Clinicians know that these are both important and work to integrate them, even when their combined interpretation is not straightforward. Similarly, in public health, policy-making practices in Italy have the opportunity to combine objective and subjective health indicators to evaluate the effectiveness of the health system from both perspectives: that of medical experts and that of the population.

Finally, we suggest that integrating SRH measures within Italian national health studies would help understand how social determinants – such as income or education – affect SRH on the Italian territory. A national health system concerned with how people feel (not just with what disease they have) would want to address social disparities in SRH, in concertation with policy-makers across other departments in the government.

## Conclusion

In this paper, we have used a subset of Italian respondents from the Health Interview Survey to look at the validity of self-rated health (SRH) as an indicator for equity analysis. We found that SRH measures were independently associated with age, chronic health conditions and functional limitations, three variables that had a summative effect on SRH. We also found that SRH could predict hospitalisation and specialist consultation better than diagnosed health conditions. Finally, we found that SRH was more significantly associated with income than actual diagnosed health conditions.

Our findings suggest that integrating SRH measures within national health studies and policy-making will help Italian health policy-makers in three ways. Firstly, SRH measures will help predict healthcare demand and allocate resources across the national healthcare system more effectively. Secondly, SRH measures will also help assess the subjective effectiveness of the treatment offered by the national healthcare system. Finally, SRH measures will help identify areas of inequity that require special attention.

A national health system able to integrate SRH measures within its indicators for policy-making will be in a better position to achieve its mission of helping people feel well. We look forward to a national health system increasingly concerned with people’s feeling of wellbeing and not simply with their diseases.

## Abbreviations

CHC: Chronic Health Conditions
FL: Functional Limitations
H: Hospitalisation
HIS: Health Interview Survey
MSC: Medical Specialist Consultations
MU: Medicine Use
PEI: Household Income
SRH: Self-Rated Health
